# Development of Metagenomic Methods for Health Monitoring of Endangered Species Using Fecal Samples

**DOI:** 10.1111/eva.70199

**Published:** 2026-01-29

**Authors:** Román Sapino, Ángel Fernández‐González, Jose Castresana

**Affiliations:** ^1^ Institute of Evolutionary Biology (CSIC‐Universitat Pompeu Fabra) Barcelona Spain; ^2^ Biosfera Consultoría Medioambiental S.L. Oviedo Spain

**Keywords:** bacteria, *Galemys pyrenaicus*, metagenomics, wildlife management

## Abstract

Metagenomic analysis of fecal samples is emerging as a powerful tool for monitoring endangered species, particularly in assessing the burden of pathogens and parasites that can threaten population viability. However, accurate identification in non‐model species remains challenging due to the frequent absence of host‐specific pathogen reference genomes. In this study, we developed a robust computational framework for detecting potentially pathogenic bacteria from metagenomic sequences by mapping them to available reference genomes in databases. Several key parameters affecting the analysis, including mapping algorithm, database configuration, and identification parameters, were analyzed to optimize detection sensitivity and specificity. Applying this approach to fresh fecal samples of the Iberian desman ( *Galemys pyrenaicus* ), a critically endangered semi‐aquatic mammal, we identified 26 potentially pathogenic bacterial species, with prevalences ranging from isolated cases to nearly half of the individuals examined. Furthermore, our analysis revealed that some desmans had atypical compositions of potential pathogens, suggesting variations in environmental exposure or host genetic factors. This work demonstrates a novel application of fecal metagenomics for species‐level detection of microorganisms implicated in disease, providing a powerful approach to gain essential insights into the health and epidemiology of endangered species and to support the development of more effective conservation strategies.

## 1 Introduction

Pathogenic and parasitic species can have a significant impact on the survival and reproduction of wild species (Daszak et al. 2000; De Castro and Bolker 2004; Smith et al. 2006; Pedersen et al. 2007; Blanchong et al. 2016). For endangered species, accurately assessing the burden and diversity of these pathogens is essential for developing effective conservation strategies. Traditional methods of studying infectious diseases often require invasive sampling techniques, which can be particularly problematic for endangered species. In this context, the analysis of fecal samples offers a powerful alternative to gather critical health information without the need for traditional sampling, making it particularly valuable for species of conservation concern (Beja‐Pereira et al. 2009; Carroll et al. 2018; Queirós et al. 2023).

Metagenomics has revolutionized our ability to profile the taxonomic composition of complex samples, including fecal samples. This advance has made it possible to study areas as diverse as diet, the microbiome, and even the host genome using only feces (Srivathsan et al. 2015, 2016; Gibson et al. 2019; Taylor et al. 2022; de Flamingh et al. 2023). Nevertheless, despite the potential of metagenomics to provide detailed insights into the presence and diversity of parasites and pathogens using fecal samples, its application to the accurate detection of these species and the health assessment of endangered species remains largely unexplored since it was first proposed (Srivathsan et al. 2015, 2016).

Several metagenomic methods are available to detect pathogenic bacteria from environmental samples such as feces. The algorithms used vary in computational efficiency and the level of detail provided for taxonomic identification of the sequences. At one end of the spectrum are rapid taxonomic profiling methods, which include k‐mer‐based tools like Kraken (Lu et al. 2022) and fast protein aligners like Diamond (Buchfink et al. 2015). Both employ lowest common ancestor strategies to provide taxonomic classification for as many sequencing reads as possible. These methods accurately identify species when reference sequences are available, or otherwise provide higher‐order classification. A significant recent advance in this category is the sylph program (Shaw and Yu 2025), which estimates the average nucleotide identity of reference genomes against a metagenome using k‐mer statistics, enabling more accurate species‐level identification than other methods in this category (Shaw and Yu 2025). These fast methods are highly effective for microbiome and large‐scale ecological studies, which often focus on broad community composition at higher taxonomic levels (Quince et al. 2017; Pinto and Bhatt 2024). Mapping‐based approaches, in contrast, are better suited for applications requiring the highest level of confidence in species‐level identification, such as clinical diagnosis and pathogen surveillance. Examples of these methods include PathoScope (Francis et al. 2013; Hong et al. 2014), SURPI (Naccache et al. 2014; Gu et al. 2021), inStrain (Olm et al. 2021), and Metapresence (Sanguineti et al. 2024). These pipelines use mapping programs like SNAP (Zaharia et al. 2011) or Bowtie2 (Langmead and Salzberg 2012) to align individual reads against reference genomes. Unlike rapid taxonomic classification methods, which provide summary statistics for species identification, mapping methods can use multiple lines of evidence derived from the genome alignment. These include the mapping quality (MAPQ) score (Li et al. 2008; Langmead 2017), which reflects the probability that a read is aligned to the correct position in the reference genome, serving as a proxy for the confidence that the read truly originates from the species genome used for mapping. Furthermore, this approach allows for a more thorough examination of alignment patterns, particularly when using complete genomes as reference. Methods such as inStrain and Metapresence have moved from simply counting mapped reads to incorporating parameters like breadth of coverage and homogeneity of read distribution. This multiple verification allows these methods to distinguish true presence from artifacts caused by conserved domains, repetitive regions or contamination (Olm et al. 2021; Sanguineti et al. 2024), providing a more robust framework for species identification. The ability to detect these artifacts is crucial when exact reference genomes are unavailable, as is often the case with non‐model species. Due to their greater computational demands, mapping‐based approaches are often applied to curated databases of target taxa to ensure tractable analysis times. Finally, assembly‐based methods can also yield high‐confidence results, but they require high coverage to assemble genomes and may only detect a fraction of the species in a sample due to the depth of sequencing required, making them less effective for low abundance species in complex microbial communities (Nurk et al. 2017; Blanco‐Miguez et al. 2023)–precisely the organisms that can be of clinical relevance as opportunistic pathogens.

Despite significant progress in methods for the accurate identification of pathogens and parasites in human health applications, there remains a notable gap in the application of these techniques to non‐model species, mainly due to the need for complete pathogen reference genomes of the exact or closely related species in databases. However, the increasing number of complete pathogen genomes now supports their use for specific applications (Goldfarb et al. 2024). Developing methods to obtain accurate pathogen identifications from feces using available genomic information is essential for enhancing disease surveillance in endangered species.

The Iberian desman ( *Galemys pyrenaicus* ) is a critically endangered semi‐aquatic mammal endemic to the Iberian Peninsula (Palmeirim and Hoffmann 1983). The species faces numerous threats, including habitat destruction, water pollution, and the presence of barriers such as reservoirs and hydroelectric power plants that disrupt its riverine habitat (Quaglietta et al. 2024). These barriers not only fragment the desman's habitat, but also isolate its populations, leading to significant inbreeding problems that further jeopardize the species' survival (Escoda et al. 2019, 2022). Despite its endangered status, there is a lack of comprehensive research on pathogens affecting the Iberian desman, with only studies using PCR‐based methods to detect pathogens in this species (Ripa et al. 2023). However, populations of this species have been declining rapidly or disappearing over the last two decades for reasons that remain largely unknown. Understanding the impact of pathogens and parasites is essential not only for the management and recovery of Iberian desman populations in situ, but also for planning any future conservation strategies, such as captive breeding and translocations, as these strategies carry the risk of inadvertently spreading diseases if the health status of the populations involved is not thoroughly understood (Sainsbury and Vaughan‐Higgins 2012; Gaywood et al. 2022). Therefore, detailed studies of the pathogens and parasites affecting the Iberian desman are critical to ensure the success of conservation efforts and prevent further population extinctions.

The aim of this study is to develop and test a robust bioinformatics pipeline based on mapping methods to identify bacterial species of clinical or veterinary relevance in fecal samples using metagenomic sequencing. Fresh samples from the Iberian desman were used to validate the method. Our approach specifically addresses the limitations of applying previous methods to non‐model species, which often lack comprehensive reference genomes of their pathogens. By focusing on bacteria, which have smaller genomes and are better represented in genomic databases, we aim to establish an efficient and reliable approach that can be used for long‐term health monitoring of microorganisms implicated in the disease of endangered species.

## 2 Materials and Methods

### 2.1 Fecal Samples Collection and DNA Extraction

Fresh fecal samples were collected in 2018 and 2019 from the Iberian desman population of the Central System, located in the center of the Iberian Peninsula. A total of 23 samples (Table S1) were obtained from four different hydrological units or subpopulations: Becedillas, Aravalle, Endrinal and Adaja. Fecal samples were collected during the capture of individuals immediately after deposition and placed in tubes with ethanol. This approach preserves DNA integrity and minimizes the risk of environmental contamination that can occur with fecal samples collected in rivers during field surveys of the Iberian desman (Hawlitschek et al. 2018; Oliveros et al. 2023). The work of capturing individuals was part of a conservation program independent of this study promoted by the Ministry of the Environment through the Biodiversity Foundation, the Duero River Basin Authority, and the Autonomous Government of Castilla y León through the Patrimonio Natural Foundation, in Spain.

DNA was extracted using the QIAamp DNA Mini Kit (QIAGEN) following the manufacturer's instructions and quantified using a Qubit fluorometer with the Qubit dsDNA High Sensitivity Assay Kit (Thermo Fisher Scientific).

### 2.2 Shotgun Metagenomic Library Construction and Sequencing

Shotgun metagenomic libraries were constructed using the NEBNext Ultra II FS DNA Library Prep Kit (New England Biolabs). Extracted DNA (26 μL per sample) was enzymatically fragmented at 37°C for 15 min. Specific Illumina adapters were ligated, and fragments of 150–250 bp insert size were selected using NEBNext Sample Purification Beads (New England Biolabs). Each sample was then indexed and amplified through 12 cycles of PCR, cleaned, and quantified using the Qubit fluorometer. Fragment size distribution (270–370 bp) was assessed by E‐gel EX 2% agarose gel electrophoresis (Invitrogen). Finally, equimolar amounts of each library were pooled and sequenced on an Illumina platform at Macrogen Inc. (South Korea) to generate 150 bp paired‐end reads.

### 2.3 Quality Control and Filtering of Endogenous Sequences

Reads of low quality, shorter than 100 bp, or duplicated, as well as adapter sequences, were filtered with FASTP version 0.23.2 (Chen 2023), while sequences with repetitive motifs were filtered with BBDUK 39.01 (https://archive.jgi.doe.gov/data‐and‐tools/software‐tools/bbtools/). The remaining reads were aligned to the reference *G. pyrenaicus* genome (Escoda and Castresana 2021) using Bowtie2 version 2.5.0 (Langmead and Salzberg 2012). The number of reads aligning to the host nuclear genome was recorded to quantify the yield of endogenous DNA for each sample. Unmapped sequences from this step were used in subsequent steps to detect pathogenic bacteria and are available in Dryad (see Data Availability Statement).

### 2.4 Reference Bacterial Genomes

Our analysis of bacterial identification methods from metagenomic sequences proceeded in two stages: an initial evaluation of methods and parameters using reference genomes of the genus *Yersinia*, followed by the application of the optimized workflow to a more comprehensive set of reference bacterial genomes.

The genus *Yersinia*, detected in initial analyses of desman samples, was chosen as a case study to assess identification methods for several reasons. Firstly, this genus comprises 26 closely related species listed in the NCBI taxonomy, providing a challenging test for accurate identification. In addition, four of these species are pathogenic (Table S2), including the well‐known plague‐causing bacterium *Y. pestis* . Therefore, the species of this genus highlight the critical need for robust methods for accurate identification to species level. Reference genomes for the *Yersinia* species were obtained from the NCBI Genome Database (https://www.ncbi.nlm.nih.gov/datasets/).

After setting the main pipeline steps and identification criteria with the *Yersinia* genus, a broader analysis was conducted using a curated list of bacterial species with pathogenic potential. To compile this list, we downloaded the reference genomes of species found in the Pathogen Detection Project of NCBI (https://www.ncbi.nlm.nih.gov/pathogens/) in February 2024, serving as our primary source. We complemented this list with additional bacterial species found in the Virulence Factor Database (VFDB, http://www.mgc.ac.cn/VFs/) (Liu et al. 2022), relevant literature concerning wildlife health (Barandika et al. 2007; Pedersen et al. 2007; Cantas and Suer 2014; White and Razgour 2020; Ali and Alsayeqh 2022; Sabour et al. 2022; Suminda et al. 2022), and online sources related to pathogens affecting wildlife in Europe and, particularly, Spain (e.g., https://ewda.org/diagnosis‐cards/, animal health section of https://www.mapa.gob.es, etc.). This compilation resulted in 137 species that included the four pathogenic *Yersinia* species mentioned above (Table S3). This list was designed to test our pipeline with a wide range of bacterial groups, but it was not intended to be an exhaustive list of all bacterial pathogenic species. It should also be noted that the list includes pathogens, but also opportunistic species that are common commensals in the mammalian gut. However, these species can become pathogenic under conditions of host stress or immunosuppression, making their detection relevant for health monitoring. *Cutibacterium acnes* was included in the initial analyses and was found in most samples, but it has been reported that this species is a likely contaminant of kits and reagents (Gu et al. 2019) and was excluded from the final analyses, which were based on 136 species.

### 2.5 Alignment of Sequences to Reference Genomes of *Yersinia* Species

The set of 26 *Yersinia* species was used to test the performance of different methods and parameters in the pipeline. First, the set of exogenous reads was aligned separately to each of the respective reference genomes using Bowtie2 (Langmead and Salzberg 2012) in the end‐to‐end alignment mode and the “‐‐sensitive” option. The end‐to‐end mode ensures that all positions of each read are involved in the alignment, without trimming or clipping. In addition, the options “‐‐no‐discordant” and “‐‐no‐mixed” were used to capture only read pairs where both reads align and have the expected orientation and distance between them. Subsequently, alignments were converted to BAM format, PCR duplicates removed, and reads with MAPQ values lower than 20 filtered using SAMtools v1.9 (Li et al. 2009). MAPQ reflects both alignment similarity and uniqueness, enabling the removal of ambiguously mapped reads. Alignments generated from single‐species databases often contained positions with extreme depth of coverage values in conserved and repetitive regions. Outlier positions were defined as those with a depth of coverage greater than the alignment mean plus three times the standard deviation, calculated in logarithmic space after excluding zero‐depth positions. Reads overlapping these positions were removed using SAMtools. Using the same program, genome alignment statistics were calculated for each sample and bacterial species, including the number of mapped reads, the mean MAPQ of the alignment, the number of positions in the reference genome sequenced with at least one read, the breadth of coverage (%; percentage of the reference genome covered by at least one read), and the depth of coverage (X; mean number of mapped reads at each genome position). The overall alignment quality was assessed by visualizing coverage across the genome in graphs constructed with Qualimap 2.2.2 (Okonechnikov et al. 2016), which represents the mean depth of coverage in 4000 windows of the genome.

Bowtie2 can be used with single‐species or multi‐species databases. For our primary species identification pipeline, a Bowtie2 database was constructed for each species. In addition, a combined database containing the set of all species was constructed to assess the effect of database configuration on analysis performance. Since there were few depth‐outlier positions in the combined databases, the step to remove them was not applied in this database configuration. When using single‐species databases, samples with multiple positive identifications within the same genus or among closely related species were further analyzed by comparing the numbers of unique and shared reads for each positive species using Venn diagrams.

The set of exogenous reads was also aligned against the *Yersinia* reference genomes using other mapping methods and conditions. Thus, we used Bowtie2 in local alignment mode, as well as another widely used DNA mapping program, BWA 0.7.17 (Li and Durbin 2009). For the latter, both the BWA‐mem and BWA‐aln algorithms were evaluated in their default configurations. The MAPQ scales are different in Bowtie2 and BWA, with a maximum of 42 for Bowtie2 and 60 for BWA. As mentioned above, a MAPQ cut‐off of 20 was used for Bowtie2. The distributions of MAPQ values for both methods in the same set of samples showed that the equivalent threshold for BWA was ~30, so this value was used to filter mapped reads with this program.

### 2.6 Species Identification

To identify potential bacterial species within fecal samples, we initially applied a filter based on breadth of coverage, retaining reference genomes with ≥ 0.25% genome coverage, which approximately corresponded to 60 reads mapped to an average bacterial genome (with a range of 14 to 140 reads, depending on the genome size).

To improve the accuracy of species identification, we applied the Metapresence program (Sanguineti et al. 2024), which calculates two key parameters, the Breadth‐Expected Breadth Ratio (BER) and the Fraction of Unexpected Gaps (FUG), to assess the homogeneity of read distribution across alignments. The BER metric is defined as the ratio of the observed to the expected breadth of coverage, where the latter is derived from the number of mapped reads under the assumption of a Poisson mapping process (Olm et al. 2021; Sanguineti et al. 2024). A BER value of 1 indicates that reads originate from the same species as the reference genome used for mapping, while lower values suggest mapping to a closely related species, which results in a reduced coverage homogeneity. Based on prior analyses of reference genomes with varying degrees of divergence, BER values around 0.8 correspond to reference genomes with approximately 98% identity to the true species, while values around 0.5 correspond to 96% identity (Sanguineti et al. 2024). In studies where well‐represented bacterial reference genomes are available, such as human samples analyses, a BER threshold of 0.8 is recommended for identification. However, given the greater divergence expected between the bacterial reference genomes and the actual species present in the Iberian desman samples, we adopted a more permissive BER threshold of 0.65, corresponding to approximately 97% identity (Sanguineti et al. 2024).

To assess alignment homogeneity in low coverage alignments (< 0.1X), where BER is less effective, we used the FUG parameter, which evaluates mapping homogeneity based on the distance between consecutive non‐paired reads (Sanguineti et al. 2024). Under a Poisson mapping model, the expected FUG value is ~0.632, with lower values indicating irregular mapping patterns. In human studies, a FUG threshold of 0.5 for both forward and reverse reads has been used for species identification (Sanguineti et al. 2024). Since this parameter is less sensitive to genome divergence, the same 0.5 threshold was used to define positive identifications in our study.

### 2.7 Alignments to Reference Genomes of Pathogenic Bacterial Species

Following the previous optimizations, Bowtie2 was used in end‐to‐end mode to align the exogenous reads from each sample to the set of 136 bacterial species using both single‐species and combined databases, with identifications based on the breadth of coverage as well as on the BER and FUG parameters as above.

Principal Component Analyses (PCA) were performed on the breadth of coverage values for all species to compare the overall health status of the Iberian desmans using the single‐species databases. A similar PCA was obtained using the combined database. As an additional test, we performed a PCA that included only bacterial species that had non‐zero coverage across all samples, yielding again similar results.

## 3 Results

### 3.1 Species‐Level Identification of *Yersinia*

**FIGURE 1:**
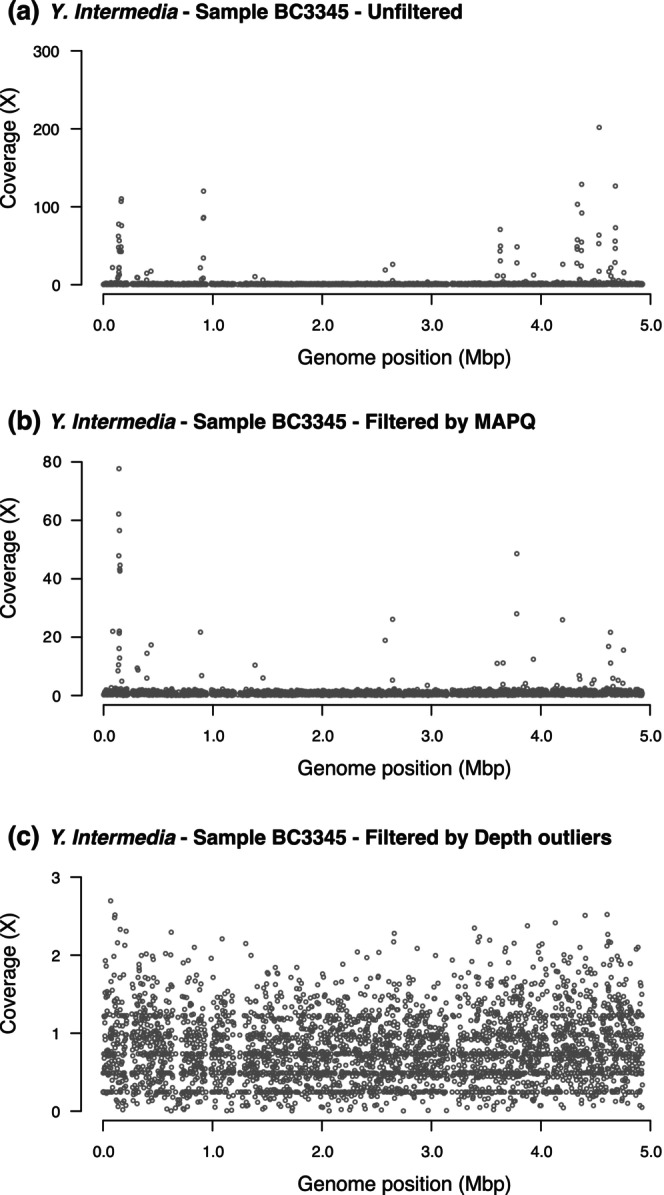
Read alignment analysis to the *Yersinia intermedia* reference genome for sample BC3345, showing depth of coverage across 4000 windows after sequential quality filtering. (a) Alignment without quality filters resulted in 47,880 mapped reads, with a mean depth of coverage of 1.45×, a breadth of 39%, and a BER of 0.53. (b) Application of a mapping quality filter (MAPQ ≥ 20) reduced the dataset to 29,610 reads, yielding a mean depth of coverage of 0.9×, a breadth of 38%, and a BER of 0.69. (c) Subsequent filtering to remove outlier depth values further reduced the number of mapped reads to 22,293, with a mean depth of 0.68×, genome coverage of 37%, and a BER of 0.83. Windows with zero coverage are not shown.

From the 23 fecal samples, we obtained between ~50 thousand and ~40 million endogenous reads, and between ~30 and ~80 million exogenous reads per sample (Table S1). To evaluate the mapping approach for identifying bacterial species, we first used the Bowtie2 aligner (end‐to‐end mode) to map exogenous reads from each sample to the genomes of 26 *Yersinia* species (Table S4), with one Bowtie2 database constructed per genome. Initial analyses showed that accurate species identification using Bowtie2 requires extensive filtering of reads to minimize misidentifications. First, reads with low MAPQ values were removed to ensure that the retained reads align confidently to the reference genome. Additionally, we observed that some genomic positions tend to accumulate an excessive number of reads either because they are highly conserved among different species in the sample or because they are repetitive; reads overlapping these outlier positions were also removed. Figure 1 illustrates how the application of these filtering steps improves an example genomic alignment.

**FIGURE 2:**
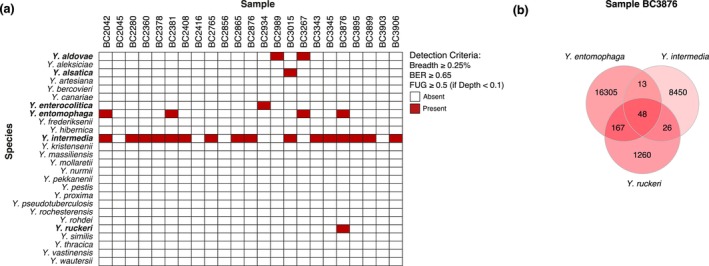
Identification of *Yersinia* species in 23 samples of Iberian desman and evaluation of read sharing of multiple species found in a single sample. (a) Species identification matrix using individual reference genomes. (b) Venn diagram comparing reads assigned to three *Yersinia* species in sample BC3876, showing shared reads (intersections) and unique reads (non‐intersecting areas) for each species.

After applying filters based on breadth of coverage, as well as the BER, and FUG Metapresence filters to the genomic alignments, we identified 25 instances of *Yersinia* species in the 23 samples (Figure 2a). *Y. intermedia* had the highest prevalence, being detected in 16 samples. The breadth of coverage for these identifications ranged from 0.27% to 45.28% of the genome (Table S4). The mean MAPQ values for the alignments of the positive identifications were close to the maximum value (ranging from 41.67 to 41.97), indicating that most reads aligned in the correct position of the reference genome used. Several samples were positive for more than one *Yersinia* species. In sample BC3876, where three *Yersinia* species were identified with high coverage, we used a Venn diagram to compare mapped reads and assess whether all three species were actually present in the sample (Figure 2b). We found that each species had a substantial number of unique reads, with minimal read sharing among them, suggesting that all three species are likely present in the sample. A similar pattern of identifications was observed when using a combined Bowtie2 database with all *Yersinia* species (Figure S1a).

### 3.2 Comparison of Mapping Tools for Species Identification

**FIGURE 3:**
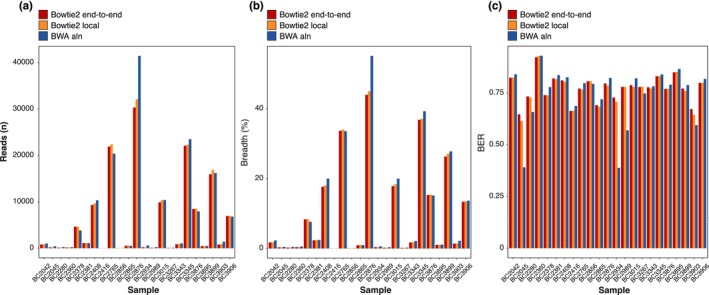
Comparison of different mapping tools (Bowtie2 end‐to‐end, Bowtie2 local, and BWA‐aln) using the *Yersinia intermedia* genome as an example, showing three key parameters for each sample. (a) Number of reads aligned. (b) Breadth of coverage. (c) BER parameter.

To verify the mappings obtained with the default Bowtie2 end‐to‐end algorithm, we compared them with those generated using Bowtie2 in local mode, BWA‐mem, and BWA‐aln. For all samples, BWA‐mem reached high mapping rates (e.g., an average of 247,151 reads aligned to *Y. intermedia* , compared to 6062, 5867, and 6449 reads for Bowtie2 end‐to‐end, Bowtie2 local, and BWA‐aln, respectively) and also higher breadth of coverage values (80% vs. 10% on average for the other methods). This was achieved mostly by reducing both read length and insert size, indicating that this mapping algorithm, in its default configuration, exhibits less stringent behavior and has no discrimination power at the species level. As for the other three algorithms, they produced comparable results in terms of reads mapped to each sample, breadth of coverage, and BER values (Figure 3 shows the results for *Y. intermedia* ). Consequently, the determination of the *Yersinia* species was similar for the three stringent mapping methods (Figure S1b,c).

### 3.3 Application to the Detection of Potentially Pathogenic Bacterial Species

**FIGURE 4:**
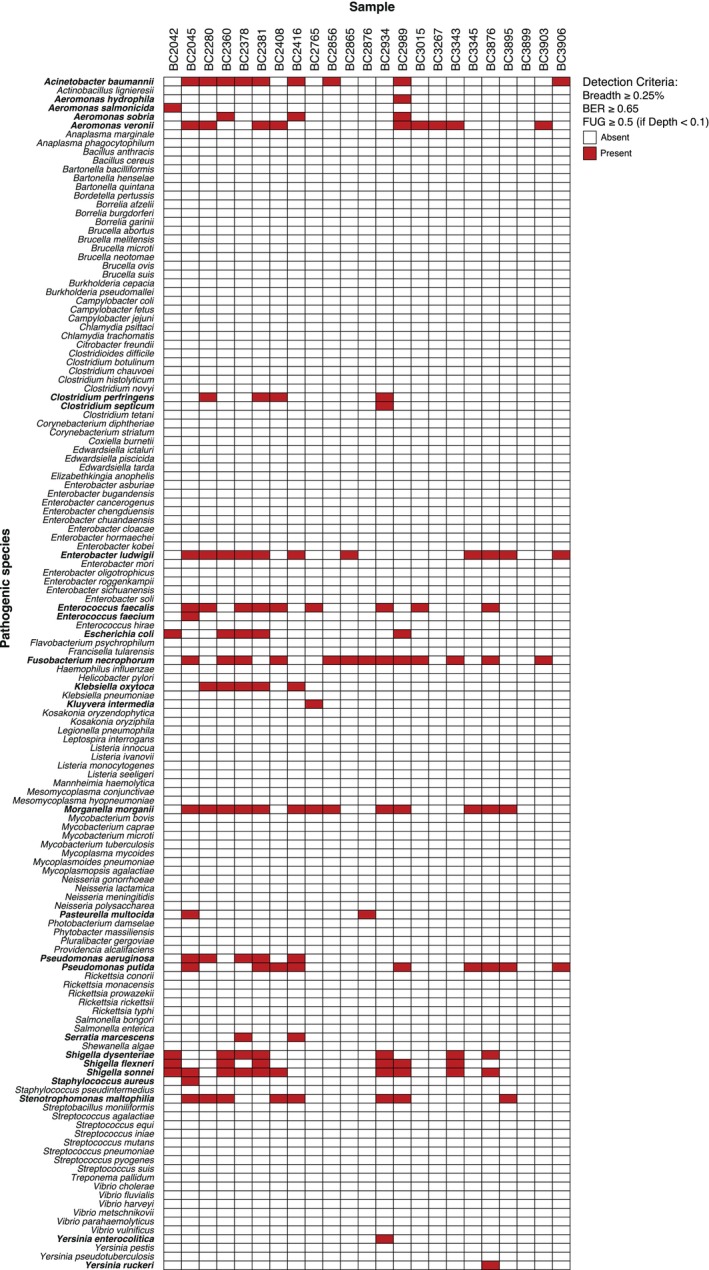
Identification matrix of 136 bacterial species in 23 samples of Iberian desman using individual reference genomes. Positive species are shown in bold for clarity.

Due to its consistent performance in the previous analysis, the Bowtie2 end‐to‐end algorithm was used with the single‐species databases for the identification of our target bacterial species. Using the identification filters as above, we determined 138 positive detections belonging to 26 different species (Figure 4). The most frequently detected species were *Fusobacterium necrophorum* and *Morganella morganii*, each found in 13 samples. Notably, all but one sample contained at least one of the species from our list. Percent coverages started at 0.26% and were as high as 91.73% for *Shigella sonnei* in one sample. Mean MAPQ values for the positive identifications were close to the maximum (ranging from 39.66 to 42). The three species of the genus *Shigella* and *Escherichia coli* , which are four very closely related species (Chattaway et al. 2017), were co‐detected in several samples. Venn diagrams for the five samples with the highest depth of coverage revealed that these species share most reads (Figure S2), suggesting that only one of the species was present in the samples. A similar identification pattern was observed for most species when the bacterial genomes were analyzed using a combined Bowtie2 database (Figure S3). However, in this configuration, only two samples were identified as positive for the *Shigella‐Escherichia* species group and with only one or two species of this group in each sample.

Table 1 provides details on a set of identified bacterial species with documented relevance in freshwater ecosystems, their associated pathologies, and wildlife species in which these microorganisms were found to cause disease.

**TABLE 1:** Bacterial species found in Iberian desman feces with documented relevance in freshwater ecosystems. Only one species per genus, the one with the highest prevalence or relevance, has been chosen as an example. For each species listed, its prevalence and a brief description of associated pathologies as found in different sources are given. In addition, examples of other relevant wildlife species in which the bacterial species has been found are given.

Bacterial species	Prevalence	Associated pathologies	Examples of relevant wildlife species where the bacterial species was found
*Acinetobacter baumannii*	9	Opportunistic infections, pneumonia, bloodstream infections, meningitis	European mink (Cano‐Terriza et al. 2017)
*Aeromonas veronii*	9	Wound infections, diarrhea, and sepsis in fish	Fish (Austin and Austin 2016); Pond turtle (Guz et al. 2021)
*Clostridium perfringens*	4	Food poisoning, gas gangrene, enteritis necroticans.	Eurasian otter (Rohner et al. 2021)
*Enterococcus faecalis*	9	In humans, urinary tract infections, bacteremia, peritonitis, endocarditis	Beaver (Laukova et al. 2015)
*Escherichia coli*	5	In humans, diarrhea, urinary tract infections, sepsis, meningitis	Eurasian otter, Pond slider, American mink (Vulfson et al. 2001; Rohner et al. 2021; Mengistu et al. 2022)
*Klebsiella oxytoca*	5	In humans, opportunistic infections	Pond slider (Mengistu et al. 2022)
*Morganella morganii*	13	In humans, post‐operative wound and urinary tract infections	Pond slider, American mink (Mengistu et al. 2022)
*Pasteurella multocida*	2	Avian cholera	Waterfowl (Blanchong et al. 2006)
*Pseudomonas aeruginosa*	5	Opportunistic infections	Fish (Ardura et al. 2013); Pond slider (Mengistu et al. 2022); American mink (Bai et al. 2023)
*Yersinia ruckeri*	1	Enteric redmouth disease in salmonids	Salmonids (Austin and Austin 2016); Fish, Muskrat (Pajdak‐Czaus et al. 2019)

### 3.4 Overall Health Assessment of the Iberian Desman Population

**FIGURE 5:**
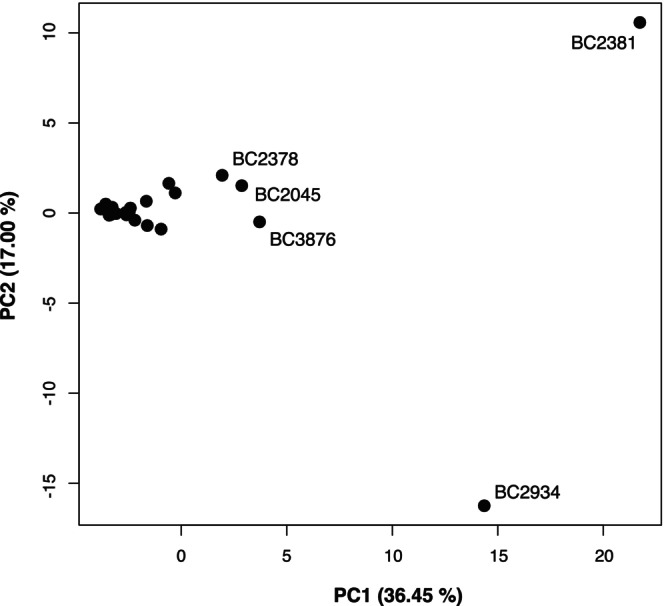
Principal Component Analysis based on breadth of coverage of the bacterial species evaluated in 23 Iberian desman samples. The sample names of outliers are indicated. The percentage of variance explained by each principal component (PC1 and PC2) is shown on the corresponding axis.

A PCA based on the breadth of coverage showed that most of the Iberian desman samples clustered together, but the analysis revealed five outliers, with two of them particularly well‐defined (Figure 5). These outliers generally had a high number of species from our list of target bacteria and a high breadth of coverage. Four of the desmans with atypical patterns were from the Endrinal, while one belonged to the Adaja hydrological unit (Table S1).

## 4 Discussion

### 4.1 Validation of a Mapping Methodology for the Detection of Potential Pathogens From Fecal Samples

In this work, we developed and validated a method to identify bacterial species of clinical and veterinary relevance from fecal samples based on the mapping of unassembled reads obtained by metagenomic sequencing to reference genomes. This approach differs fundamentally from methods designed to profile the general composition of the microbiome, such as Kraken (Lu et al. 2022), Diamond (Buchfink et al. 2015), or sylph (Shaw and Yu 2025). Instead, it is more similar to pathogen detection methods used in human disease studies, where the primary objective is species‐level identification of target pathogens, such as PathoScope (Francis et al. 2013; Hong et al. 2014), SURPI (Naccache et al. 2014; Gu et al. 2021), or Metapresence (Sanguineti et al. 2024). To our knowledge, no studies have systematically applied these species‐level pathogen detection methods to endangered wildlife species. The effectiveness of our pipeline for wildlife health monitoring was demonstrated by its ability to identify potentially pathogenic bacterial species in fecal samples from the Iberian desman. Several factors affecting the identifications were systematically analyzed to better understand their impact, optimize the method, and guide their application under different computational conditions.

We showed that different strict mapping algorithms like Bowtie2 (in two different modes: local and end‐to‐end) and BWA‐aln consistently produced similar mapping results and thus similar species identifications despite their algorithmic differences, reinforcing confidence in their ability to provide accurate taxonomic classifications.

For initial species identifications, we used breadth of genome coverage, as recommended in recent studies (Olm et al. 2021; Sanguineti et al. 2024), rather than the more traditional reliance on the proportion of mapped reads (Francis et al. 2013; Hong et al. 2014; Naccache et al. 2014; Gu et al. 2021). This approach helps avoid false positives caused by highly conserved regions, repetitive sequences, or contamination of reference genomes with sequences from other species (Treangen and Salzberg 2011; Steinegger and Salzberg 2020). However, while breadth of coverage is superior to depth‐based metrics, it alone is insufficient for reliable identification, as it does not distinguish between reads distributed randomly across the genome and those clustered in a few specific regions. The recent introduction of the BER and FUG parameters of the Metapresence program to evaluate the homogeneity of read distribution within reference genomes is a significant improvement, as they allow species identification even when a small number of reads are mapped (Sanguineti et al. 2024). In addition, applying quality filters to the alignments, such as those based on MAPQ and the removal of depth outliers, helps minimize the influence of spurious reads on BER and FUG parameters, which could otherwise lead to false negatives, particularly when exact reference genomes are not available (Figure 1). Although the recommendation of the authors of the Metapresence program was to consider alignments with more than 80 mapped reads, we used a breadth of coverage threshold instead, as it can be better adapted to genomes of different sizes or even to genomic fragments. Our threshold of 0.25% breadth of coverage corresponds to approximately 60 mapped reads for an average‐sized bacterial genome, a value comparable to the threshold of 80 mapped reads used in Sanguineti et al. (2024). For our set of reference genomes (Table S3), this minimum breadth corresponds to a range of approximately 14 to 140 reads, ensuring that a consistent threshold is applied to all species, regardless of their genome size. The combined use of these filters enabled the removal of non‐homogeneous alignments, facilitating the identification of species or their closest relatives present in the sample. Without BER and FUG, ensuring reliable species identification would require much higher breadth of coverage thresholds (2%–3%), which would significantly reduce sensitivity. However, the most appropriate threshold may vary depending on the specific conditions of individual studies. For instance, when analyzing highly pathogenic or other relevant species, samples with lower breadth of coverage or number of mapped reads should be considered for further investigation to ensure comprehensive monitoring.

The type of reference database to be used with the mapping algorithm is another critical factor, particularly for very closely related species. Our results with the *Shigella* and *Escherichia* species group (Figure 4, Figures S2 and S3) showed that unspecific mappings to closely related species in the database may be higher when using a single database for each genome analyzed, leading to more shared reads between them. In contrast, using a combined database for all genomes may result in the loss of potential positives due to ambiguous mappings with low mapping quality values. In essence, single‐species databases typically provide greater sensitivity in closely related species because they focus on detecting specific bacteria without competition with sequences from other species, whereas combined databases improve specificity by offering a broader reference to discriminate between similar species, although they may reduce sensitivity due to ambiguous or low‐confidence mappings to multiple species. Therefore, case‐specific validation should be conducted to determine the most suitable database and detection criteria for each species under study to ensure that the method provides robust identifications.

### 4.2 Implications for the Conservation of the Iberian Desman

Understanding pathogen burden and diversity in endangered species is critical to inform management strategies, particularly in the context of conservation efforts such as captive breeding and translocation programs (Sainsbury and Vaughan‐Higgins 2012; Gaywood et al. 2022). In our study, we identified 26 species of potentially pathogenic bacteria in 23 Iberian desman fecal samples, with varying levels of genome coverage and prevalence. The sample size was very limited, which prevents any generalization of the results. Undoubtedly, a larger sample size, covering a wider geographical area and different time points, would provide a more comprehensive understanding of pathogen prevalence and diversity in this mammal. Although the main focus of the work was methodological, with the aim of developing a strategy that can be used for health monitoring, this pilot analysis also provided essential baseline data on the presence and diversity of pathogens in the Iberian desman.

Some of the bacteria detected are certainly part of the normal intestinal microbiome, which typically cause disease only in the event of some type of immunological weakness of the animal. However, other species found are more specific to the unique semi‐aquatic niche of the desman. As shown in Table 1, many of the identified species are known to cause infections in fish (Ardura et al. 2013; Austin and Austin 2016; Pajdak‐Czaus et al. 2019) and other aquatic or semi‐aquatic mammals, including European mink, American mink, Eurasian otter, beaver, muskrat, pond slider, and pond turtle (Vulfson et al. 2001; Laukova et al. 2015; Cano‐Terriza et al. 2017; Pajdak‐Czaus et al. 2019; Guz et al. 2021; Rohner et al. 2021; Mengistu et al. 2022; Bai et al. 2023) as well as waterfowl (Blanchong et al. 2006). In some cases, these infections have been associated with disease or mortality in these animals, underscoring the potential severity of some of these pathogens. The sharing of pathogens among aquatic species may be explained by the fact that the aquatic environment can facilitate the transmission of infections, spreading pathogens over a wide area and potentially increasing exposure to multiple microbial threats (Cabral 2010). Thus, these results highlight the complex and potentially pathogenic microbial landscape to which the Iberian desman is exposed.

The PCA (Figure 5) revealed substantial variability in bacterial patterns among Iberian desmans, suggesting differences in the composition of pathogenic or opportunistic bacteria, which may be highly relevant for assessing the health status of the analyzed individuals. In particular, five desmans emerged as clear outliers in the PCA plot, indicating atypical microbial signatures. No specific bacteria, but rather a combination of them, seems to be driving the existence of these desman outliers. It remains unclear whether these desmans exhibited these profiles by chance, due to a compromised immune system potentially related to inbreeding, or as a result of specific environmental conditions. The geographic distribution of the outlier desmans may provide a clue, as four of them belonged to the same hydrological unit, Endrinal, so that 40% of the desmans in this subpopulation would have an elevated or altered composition of potential pathogens. The Endrinal river supports a significant livestock density (personal observation of the authors), which could be behind the unique patterns of potential pathogens found in the desmans of this river. However, additional sampling and detailed environmental analyses in this and other hydrological units are needed to better understand the origin of these pathogens and opportunistic bacteria, and the factors driving their altered presence in some individuals and populations.

Future studies of Iberian desman pathogens should include different populations across the species' range and throughout the year to understand population health trends and to prioritize conservation efforts to the most vulnerable populations. Integrating in these studies the analysis of genetic factors, such as inbreeding and mutational load, will be crucial for understanding their influence on pathogen load and susceptibility. Our results demonstrate that this metagenomic workflow can simultaneously recover both exogeneous and host DNA, and that the endogenous yield in some samples is sufficient to make such population genomic analyses feasible (Table S1). Furthermore, epidemiological studies should be a priority to understand how these pathogens are transmitted, for example through water or through contact with other wildlife species, to identify potential transmission hotspots, such as excessive livestock densities in mountainous areas or wastewater discharge points near human settlements, and to assess their potential impact on the health and viability of Iberian desman populations.

### 4.3 Method Challenges and Prospects

This study represents a significant advance in pathogen detection methods for endangered species by providing a novel and effective approach for monitoring microbial diseases. Although this study was based on fecal samples obtained from captured specimens, the approach can be applied to non‐invasive samples obtained from rivers, provided they are sufficiently fresh. To demonstrate the effectiveness of the method, we used a dataset of 136 bacterial reference genomes for mapping. We focused on bacteria because of their relatively good representation of complete genomes in databases, as well as their reduced genome sizes, which allowed us to test a wide variety of parameters and analysis conditions with reasonable computational effort. Viral and eukaryotic pathogens, on the other hand, present different challenges for metagenomic detection, which were beyond the scope of this study. Future research should address these challenges and optimize bioinformatic tools for detecting such pathogens, as they can also have a significant impact on the overall health and viability of wildlife species (Nunn and Altizer 2005; Pedersen et al. 2007).

The dependence on reference genome databases is a major limitation of mapping methods, particularly for less studied species such as the Iberian desman. Our results showed that a significant number of bacterial species with pathogenic potential (26 out of 136 analyzed) can be found in the Iberian desman using currently available databases. Many of these bacteria are known to occur in humans and other mammals, indicating that they are generalist bacteria with a wide host range for these pathogens (Shaw et al. 2020). Since specific bacterial pathogens of the Iberian desman have not yet been sequenced, they cannot be detected using this bioinformatics method; however, given an adequate parametrization of the methods used, closely related species present in databases can be detected. Eukaryotic parasite species, however, tend to be more host‐specific (Poulin 2006), so it remains to be seen whether genomes currently present in genome databases, usually isolated from humans or model species, will be useful for detecting eukaryotic pathogens in the Iberian desman or other endangered mammals. In the long term, the generation of complete reference genomes for a wide range of relevant pathogenic and parasitic species will be key to advancing the detection and assessment of pathogens in wildlife, especially in critically endangered species.

Accurate identification of pathogens is not only critical in conservation biology, but also an integral part of the One Health approach, which emphasizes the important interrelationships that exist between human, wildlife, livestock, and environmental health (Destoumieux‐Garzon et al. 2018; White and Razgour 2020). By using advanced metagenomic technologies, this study provides a reliable and potentially non‐invasive method to monitor pathogens that may pose risks, not only to wildlife and endangered species, but also to humans. This underscores the importance of further research in this area, particularly to improve genomic tools and the representation of reference genomes of wildlife pathogens and parasites in databases, thereby contributing to the broader goal of protecting endangered species and maintaining ecosystem health.

## Disclosure

Benefits Generated: Benefits from this research accrue from the sharing of our data and results on public databases as described above.

## Conflicts of Interest

The authors declare no conflicts of interest.

## Supporting information


**Data S1:** eva70199‐sup‐0001‐Supinfo.pdf.

## Data Availability

The sequence data used in this study (in fastq format), along with a document detailing the bioinformatic programs and Unix commands employed in the pipeline, are available in Dryad (https://doi.org/10.5061/dryad.p5hqbzm00).
