# Supplementary material for: Development of Metagenomic Methods for Health Monitoring of Endangered Species Using Fecal Samples
Source: Evol Appl. 2026 Jan 29;19(2):e70199. doi: 10.1111/eva.70199 (PMC12855166; doi:10.1111/eva.70199)
Supplement: Supplementary file 1 — Data S1: eva70199‐sup‐0001‐Supinfo.pdf. [file EVA-19-e70199-s001.pdf]

Supplementary Information

Development of metagenomic methods for health monitoring of endangered species using fecal samples

Román Sapino [1], Ángel Fernández-González [2], Jose Castresana [1]

[1] Institute of Evolutionary Biology (CSIC-Universitat Pompeu Fabra), Barcelona, Spain

[2] Biosfera Consultoría Medioambiental S.L., Oviedo, Spain

Corresponding author: Jose Castresana

Email: jose.castresana@csic.es

Table S1. Samples used in this study, hydrological unit or population of origin, and number of endogenous and exogenous reads for each sample.

Sample number	Sample	Hydrological unit	Endogenous reads	Exogenous reads
1	BC2042	Aravalle	47,582	61,736,980
2	BC2045	Endrinal	12,092,536	50,124,430
3	BC2280	Adaja	17,004,552	59,649,976
4	BC2360	Adaja	1,019,544	56,053,206
5	BC2378	Endrinal	12,598,510	48,532,104
6	BC2381	Endrinal	220,114	60,256,352
7	BC2408	Aravalle	2,862,668	77,905,478
8	BC2416	Adaja	2,382,304	66,786,794
9	BC2765	Becedillas	6,056,388	47,017,792
10	BC2856	Becedillas	197,424	81,444,940
11	BC2865	Endrinal	38,876,690	30,409,082
12	BC2876	Aravalle	539,906	65,926,618
13	BC2934	Adaja	876,468	72,800,924
14	BC2989	Becedillas	362,066	63,910,586
15	BC3015	Aravalle	1,132,576	41,132,864
16	BC3267	Aravalle	816,462	75,036,328
17	BC3343	Aravalle	12,710,970	41,438,942
18	BC3345	Endrinal	10,573,752	53,654,356
19	BC3876	Endrinal	151,170	78,895,038
20	BC3895	Endrinal	1,832,636	66,061,256
21	BC3899	Endrinal	11,314,712	29,719,132
22	BC3903	Endrinal	4,590,572	43,476,990
23	BC3906	Endrinal	3,374,458	56,744,902

Table S2. Species of the genus *Yersinia* according to NCBI Taxonomy, along with the size of the reference genome used. Pathogenicity is indicated with an X.

**Species**	**Genome size (bp)**	**Source**	**Pathogenicity**
Yersinia aldovae	4,471,090	NCBI Taxonomy	
Yersinia aleksiciae	4,526,044	NCBI Taxonomy	
Yersinia alsatica	4,901,396	NCBI Taxonomy	
Yersinia artesiana	4,520,664	NCBI Taxonomy	
Yersinia bercovieri	4,428,721	NCBI Taxonomy	
Yersinia canariae	4,710,154	NCBI Taxonomy	
Yersinia enterocolitica	4,548,822	NCBI Taxonomy	X
Yersinia entomophaga	4,275,406	NCBI Taxonomy	
Yersinia frederiksenii	4,941,872	NCBI Taxonomy	
Yersinia hibernica	4,803,435	NCBI Taxonomy	
Yersinia intermedia	4,928,910	NCBI Taxonomy	
Yersinia kristensenii	4,733,508	NCBI Taxonomy	
Yersinia massiliensis	5,050,276	NCBI Taxonomy	
Yersinia mollaretii	4,603,534	NCBI Taxonomy	
Yersinia nurmii	4,143,134	NCBI Taxonomy	
Yersinia pekkanenii	5,046,986	NCBI Taxonomy	
Yersinia pestis	4,658,411	NCBI Taxonomy	X
Yersinia proxima	4,616,898	NCBI Taxonomy	
Yersinia pseudotuberculosis	4,839,430	NCBI Taxonomy	X
Yersinia rochesterensis	4,448,883	NCBI Taxonomy	
Yersinia rohdei	4,372,253	NCBI Taxonomy	
Yersinia ruckeri	3,894,226	NCBI Taxonomy	X
Yersinia similis	4,964,409	NCBI Taxonomy	
Yersinia thracica	4,420.333	NCBI Taxonomy	
Yersinia vastinensis	4,540,093	NCBI Taxonomy	
Yersinia wautersii	4,858,848	NCBI Taxonomy	

Table S3. Species of pathogenic bacteria analyzed and the size of their reference genomes.

Species	Synonym	Genome size (bp)
Acinetobacter baumannii		3,980,230
Actinobacillus lignieresii		2,236,520
Aeromonas hydrophila		4,733,702
Aeromonas salmonicida		4,954,811
Aeromonas sobria		4,683,669
Aeromonas veronii		4,561,870
Anaplasma marginale		1,202,435
Anaplasma phagocytophilum		1,481,598
Bacillus anthracis		5,503,926
Bacillus cereus		5,836,971
Bartonella bacilliformis		1,411,655
Bartonella henselae		1,905,383
Bartonella quintana		1,588,683
Bordetella pertussis		4,088,701
Borrelia afzelii		906,136
Borrelia burgdorferi		1,321,434
Borrelia garinii		905,692
Brucella abortus		3,278,307
Brucella melitensis		3,294,931
Brucella microti		3,337,369
Brucella neotomae		3,329,628
Brucella ovis		3,275,590
Brucella suis		3,315,175
Burkholderia cepacia		8,366,868
Burkholderia pseudomallei		7,085,397
Campylobacter coli		1,716,536
Campylobacter fetus		1,804,582
Campylobacter jejuni		1,641,481
Chlamydia psittaci		1,179,220
Chlamydia trachomatis		1,042,519
Citrobacter freundii		5,171,093
Clostridioides difficile	*Clostridium difficile*	4,095,894
Clostridium botulinum		3,903,260
Clostridium chauvoei		2,889,569
Clostridium histolyticum		2,740,791
Clostridium novyi		2,499,078
Clostridium perfringens		3,275,424
Clostridium septicum		3,404,718
Clostridium tetani		2,873,333
Corynebacterium diphtheriae		2,406,896
Corynebacterium striatum		2,904,831
Coxiella burnetii		2,032,807
Edwardsiella ictaluri		3,844,237
Edwardsiella piscicida		3,819,771
Edwardsiella tarda		3,720,168
Elizabethkingia anophelis		4,058,311

Enterobacter asburiae		4,768,325
Enterobacter bugandensis		4,734,039
Enterobacter cancerogenus		4,736,684
Enterobacter chengduensis		5,218,125
Enterobacter chuandaensis		4,634,324
Enterobacter cloacae		5,023,439
Enterobacter hormaechei		4,855,498
Enterobacter kobei		4,773,801
Enterobacter ludwigii		4,952,770
Enterobacter mori		4,844,012
Enterobacter oligotrophicus		4,476,585
Enterobacter roggenkampii		4,899,997
Enterobacter sichuanensis		4,711,389
Enterobacter soli		5,012,132
Enterococcus faecalis		2,870,381
Enterococcus faecium		2,919,198
Enterococcus hirae		2,845,651
Escherichia coli		5,594,605
Flavobacterium psychrophilum		2,830,557
Francisella tularensis		1,870,206
Fusobacterium necrophorum		2,288,480
Haemophilus influenzae		1,846,259
Helicobacter pylori		1,624,458
Klebsiella oxytoca		5,879,076
Klebsiella pneumoniae		5,682,322
Kluyvera intermedia		4,938,529
Kosakonia oryzendophytica		5,132,919
Kosakonia oryziphila		4,814,900
Legionella pneumophila		3,504,074
Leptospira interrogans		4,630,763
Listeria innocua		2,922,148
Listeria ivanovii		2,919,548
Listeria monocytogenes		2,944,528
Listeria seeligeri		2,797,636
Mannheimia haemolytica		2,757,078
Mesomycoplasma conjunctivae	*Mycoplasma conjunctivae*	846,214
Mesomycoplasma hyopneumoniae	*Mycoplasma hyopneumoniae*	921,093
Morganella morganii		3,906,921
Mycobacterium bovis	*M. tuberculosis variant bovis*	4,411,814
Mycobacterium caprae	*M. tuberculosis variant caprae*	4,324,961
Mycobacterium microti	*M. tuberculosis variant microti*	4,369,915
Mycobacterium tuberculosis		4,411,532
Mycoplasma mycoides		1,084,586
Mycoplasmoides pneumoniae	*Mycoplasma pneumoniae*	823,017
Mycoplasmopsis agalactiae	*Mycoplasma agalactiae*	916,806
Neisseria gonorrhoeae		2,171,755
Neisseria lactamica		2,200,224
Neisseria meningitidis		2,157,444
Neisseria polysaccharea		2,029,584

Pasteurella multocida	2,335,516
Photobacterium damselae	4,427,003
Phytobacter massiliensis	5,003,540
Pluralibacter gergoviae	5,408,082
Providencia alcalifaciens	3,990,106
Pseudomonas aeruginosa	6,264,404
Pseudomonas putida	6,156,701
Rickettsia conorii	1,268,755
Rickettsia monacensis	1,353,450
Rickettsia prowazekii	1,109,804
Rickettsia rickettsii	1,257,710
Rickettsia typhi	1,112,372
Salmonella bongori	4,773,537
Salmonella enterica	4,951,383
Serratia marcescens	5,238,537
Shewanella algae	4,990,025
Shigella dysenteriae	5,192,674
Shigella flexneri	4,828,820
Shigella sonnei	4,762,774
Staphylococcus aureus	2,821,361
Staphylococcus pseudintermedius	2,629,596
Stenotrophomonas maltophilia	4,481,118
Streptobacillus moniliformis	1,673,280
Streptococcus agalactiae	2,079,123
Streptococcus equi	2,154,778
Streptococcus iniae	2,078,360
Streptococcus mutans	2,028,032
Streptococcus pneumoniae	2,154,362
Streptococcus pyogenes	1,746,380
Streptococcus suis	2,170,808
Treponema pallidum	1,139,330
Vibrio cholerae	4,138,412
Vibrio fluvialis	4,827,733
Vibrio harveyi	5,869,008
Vibrio metschnikovii	3,831,334
Vibrio parahaemolyticus	5,165,770
Vibrio vulnificus	5,117,890
Yersinia enterocolitica	4,548,822
Yersinia pestis	4,658,411
Yersinia pseudotuberculosis	4,839,430
Yersinia ruckeri	3,894,226

Table S4. Number of reads and breadth of coverage (%) obtained from aligning reads with *Yersinia* species genomes using Bowtie2 in end-to-end mode with default parameters.

Number of Reads	BC2042	BC2045	BC2280	BC2360	BC2378	BC2381	BC2408	BC2416	BC2765	BC2856	BC2865	BC2876	BC2934	BC2989	BC3015	BC3267	BC3343	BC3345	BC3876	BC3895	BC3899	BC3903	BC3906
Y. aldovae	86	100	18	22	92	182	238	6	209	10	44	435	213	135	202	126	58	13790	296	32	263	11943	76
Y. aleksiciae	278	4048	1470	1120	1718	7118	1729	60	1371	46	1502	721	12538	1792	2490	92	266	20016	5455	148	1211	563	384
Y. alsatica	50	90	42	20	88	70	191	0	265	8	44	528	332	88	1135	10	24	5636	255	2	227	406	92
Y. artesiana	298	4600	1664	1256	1970	7798	3200	68	2561	56	1676	849	14842	1958	1184	98	286	19082	1877	150	1244	730	426
Y. bercovieri	282	4308	1542	1204	1826	7528	2347	62	1239	50	1546	671	13418	1782	2610	98	268	19044	6850	148	1302	554	364
Y. canariae	24	34	70	10	69	76	197	0	228	4	30	461	64	56	219	8	30	5700	311	6	216	390	84
Y. enterocolitica	38	84	54	20	109	82	206	4	284	6	34	577	1060	40	299	14	34	6352	299	8	300	967	109
Y. entomophaga	250	110	50	18	142	396	188	0	114	4	24	202	446	60	175	112	28	2494	16533	0	80	122	38
Y. frederiksenii	36	126	92	32	98	119	225	12	263	6	32	478	454	72	277	18	22	6310	261	6	241	386	82
Y. hibernica	24	284	50	30	78	68	166	2	204	4	36	353	300	38	208	12	32	5876	231	4	180	324	70
Y. intermedia	812	214	140	157	4648	1133	9624	14	22385	24	562	32100	204	99	10368	76	878	22293	8537	452	16872	864	6981
Y. kristensenii	40	44	20	12	54	80	199	6	206	4	24	428	57	36	195	72	26	8670	350	2	192	401	84
Y. massiliensis	286	4582	1706	1318	1944	7540	3188	62	2588	54	1586	1342	14250	1856	2702	100	278	19978	7410	156	1518	479	368
Y. mollaretii	28	64	30	8	82	61	201	6	255	6	24	468	81	54	208	10	32	4878	258	2	226	326	94
Y. nurmii	304	5234	1842	1368	1916	7864	2946	62	2550	50	1540	2546	15304	2078	2564	102	264	14782	1773	146	1478	316	334
Y. pekkanenii	300	4324	1544	1178	1810	7500	2862	64	1400	50	1632	686	13532	2006	2584	102	256	19672	6938	142	1242	699	374
Y. pestis	28	74	38	14	56	42	174	4	110	4	22	175	114	44	126	6	24	4816	147	0	138	200	42
Y. proxima	290	4754	1610	1284	1894	7750	3192	64	2165	56	1592	776	14136	1908	681	100	282	19378	5296	154	1606	829	412
Y. pseudotuberculosis	26	62	38	20	76	62	119	6	110	4	32	186	720	40	122	6	24	6172	237	0	146	202	46
Y. rochesterensis	26	86	50	28	78	106	193	6	241	2	26	430	100	26	184	10	22	5282	264	2	207	377	82
Y. rohdei	24	102	36	30	80	74	161	6	256	4	28	437	370	48	223	12	28	4880	279	6	234	396	83
Y. ruckeri	88	110	26	10	100	64	154	2	78	4	20	203	158	62	136	20	78	2412	1501	2	90	138	40
Y. similis	34	48	12	4	56	44	200	4	122	4	28	220	46	50	120	4	16	4686	168	0	120	200	38
Y. thracica	276	3784	1406	1086	1696	7498	2876	60	1262	42	1610	791	12316	1786	2230	94	268	19714	2837	138	863	599	352
Y. vastinensis	308	5076	1772	1332	1978	7840	3090	58	1877	56	1778	978	15352	2104	2724	96	284	18480	7754	156	1608	510	382
Y. wautersii	182	2970	1040	898	1308	5658	829	48	2142	38	932	658	9926	1258	1452	62	150	13868	956	82	992	357	218

Breadth of Coverage	BC2042	BC2045	BC2280	BC2360	BC2378	BC2381	BC2408	BC2416	BC2765	BC2856	BC2865	BC2876	BC2934	BC2989	BC3015	BC3267	BC3343	BC3345	BC3876	BC3895	BC3899	BC3903	BC3906
Y. aldovae	0.19	0.10	0.04	0.05	0.16	0.16	0.49	0.01	0.36	0.02	0.08	0.76	0.25	0.32	0.36	0.27	0.13	1.50	0.48	0.08	0.44	18.89	0.16
Y. aleksiciae	0.13	0.16	0.14	0.11	0.25	0.22	0.47	0.06	0.51	0.06	0.13	0.81	0.26	0.18	0.45	0.09	0.15	0.96	0.50	0.08	0.47	0.66	0.26
Y. alsatica	0.10	0.07	0.05	0.03	0.15	0.09	0.32	0.00	0.44	0.01	0.03	0.86	0.14	0.10	2.22	0.02	0.05	0.88	0.40	0.00	0.37	0.56	0.18
Y. artesiana	0.17	0.19	0.12	0.12	0.28	0.41	0.50	0.07	0.60	0.06	0.14	0.84	0.54	0.17	0.62	0.11	0.15	1.12	0.73	0.09	0.52	0.99	0.28
Y. bercovieri	0.14	0.15	0.13	0.12	0.25	0.22	0.51	0.06	0.55	0.06	0.13	0.78	0.22	0.17	0.46	0.10	0.13	0.95	0.49	0.08	0.49	0.61	0.26
Y. canariae	0.05	0.05	0.04	0.01	0.12	0.12	0.32	0.00	0.39	0.01	0.04	0.77	0.12	0.06	0.38	0.02	0.07	0.95	0.49	0.01	0.36	0.55	0.17
Y. enterocolitica	0.08	0.07	0.11	0.03	0.18	0.13	0.37	0.01	0.50	0.02	0.06	0.90	2.30	0.06	0.53	0.03	0.08	1.19	0.45	0.02	0.50	1.53	0.23
Y. entomophaga	0.66	0.09	0.06	0.02	0.27	0.97	0.24	0.00	0.17	0.01	0.04	0.32	0.14	0.10	0.32	0.27	0.06	0.40	30.47	0.00	0.13	0.20	0.08
Y. frederiksenii	0.06	0.07	0.06	0.04	0.16	0.14	0.33	0.02	0.42	0.01	0.03	0.72	0.15	0.09	0.44	0.04	0.04	0.96	0.35	0.01	0.39	0.52	0.16
Y. hibernica	0.04	0.13	0.04	0.05	0.14	0.10	0.27	0.00	0.33	0.01	0.05	0.56	0.19	0.06	0.33	0.03	0.07	0.80	0.33	0.01	0.30	0.46	0.14
Y. intermedia	1.78	0.34	0.27	0.39	8.41	2.41	18.15	0.02	34.24	0.05	0.99	45.28	0.38	0.20	18.50	0.16	1.79	37.35	15.52	1.02	27.31	1.45	13.53
Y. kristensenii	0.08	0.04	0.03	0.03	0.10	0.13	0.31	0.01	0.36	0.01	0.04	0.72	0.12	0.08	0.35	0.05	0.06	0.80	0.46	0.00	0.32	0.57	0.18
Y. massiliensis	0.12	0.13	0.10	0.09	0.18	0.15	0.32	0.05	0.33	0.05	0.11	0.55	0.18	0.13	0.31	0.09	0.12	0.73	0.36	0.06	0.31	0.43	0.19
Y. mollaretii	0.06	0.07	0.06	0.02	0.14	0.11	0.35	0.01	0.44	0.01	0.04	0.81	0.12	0.09	0.36	0.02	0.07	0.88	0.37	0.00	0.40	0.48	0.20
Y. nurmii	0.19	0.18	0.15	0.11	0.20	0.28	0.27	0.06	0.23	0.05	0.13	0.35	0.20	0.20	0.29	0.12	0.13	0.48	3.26	0.07	0.22	0.28	0.15
Y. pekkanenii	0.16	0.13	0.10	0.09	0.19	0.18	0.41	0.06	0.49	0.05	0.11	0.80	0.22	0.15	0.40	0.08	0.11	0.98	0.40	0.07	0.44	0.70	0.25
Y. pestis	0.06	0.06	0.03	0.02	0.09	0.07	0.21	0.01	0.18	0.01	0.03	0.30	0.09	0.07	0.18	0.01	0.05	0.55	0.20	0.00	0.19	0.30	0.08
Y. proxima	0.15	0.16	0.12	0.15	0.24	0.21	0.43	0.06	0.53	0.06	0.14	0.84	0.48	0.17	1.16	0.10	0.15	1.09	0.55	0.07	0.47	1.20	0.27
Y. pseudotuberculosis	0.05	0.05	0.03	0.03	0.10	0.09	0.17	0.01	0.17	0.01	0.04	0.30	0.69	0.06	0.18	0.01	0.05	0.57	0.22	0.00	0.19	0.29	0.08
Y. rochesterensis	0.06	0.06	0.04	0.04	0.12	0.17	0.35	0.01	0.41	0.00	0.04	0.78	0.16	0.06	0.32	0.02	0.05	0.88	0.41	0.00	0.38	0.61	0.18
Y. rohdei	0.05	0.07	0.04	0.04	0.12	0.12	0.30	0.01	0.43	0.01	0.04	0.75	0.16	0.08	0.39	0.02	0.07	0.93	0.41	0.02	0.41	0.69	0.18
Y. ruckeri	0.25	0.08	0.03	0.01	0.17	0.14	0.21	0.00	0.13	0.01	0.04	0.41	0.15	0.11	0.25	0.05	0.21	0.57	3.56	0.00	0.17	0.23	0.09
Y. similis	0.07	0.04	0.02	0.01	0.09	0.07	0.25	0.01	0.19	0.01	0.04	0.32	0.08	0.05	0.18	0.01	0.04	0.55	0.19	0.00	0.19	0.28	0.07
Y. thracica	0.15	0.14	0.11	0.11	0.22	0.24	0.47	0.06	0.53	0.05	0.11	0.80	0.20	0.18	0.49	0.09	0.16	1.03	0.55	0.08	0.57	0.70	0.26
Y. vastinensis	0.15	0.15	0.14	0.12	0.26	0.23	0.44	0.05	0.56	0.06	0.14	0.80	0.22	0.19	0.47	0.10	0.15	0.97	0.53	0.07	0.46	0.56	0.26
Y. wautersii	0.13	0.15	0.12	0.12	0.21	0.28	0.35	0.04	0.44	0.04	0.10	0.41	0.20	0.14	0.27	0.06	0.10	0.70	0.42	0.05	0.27	0.39	0.15

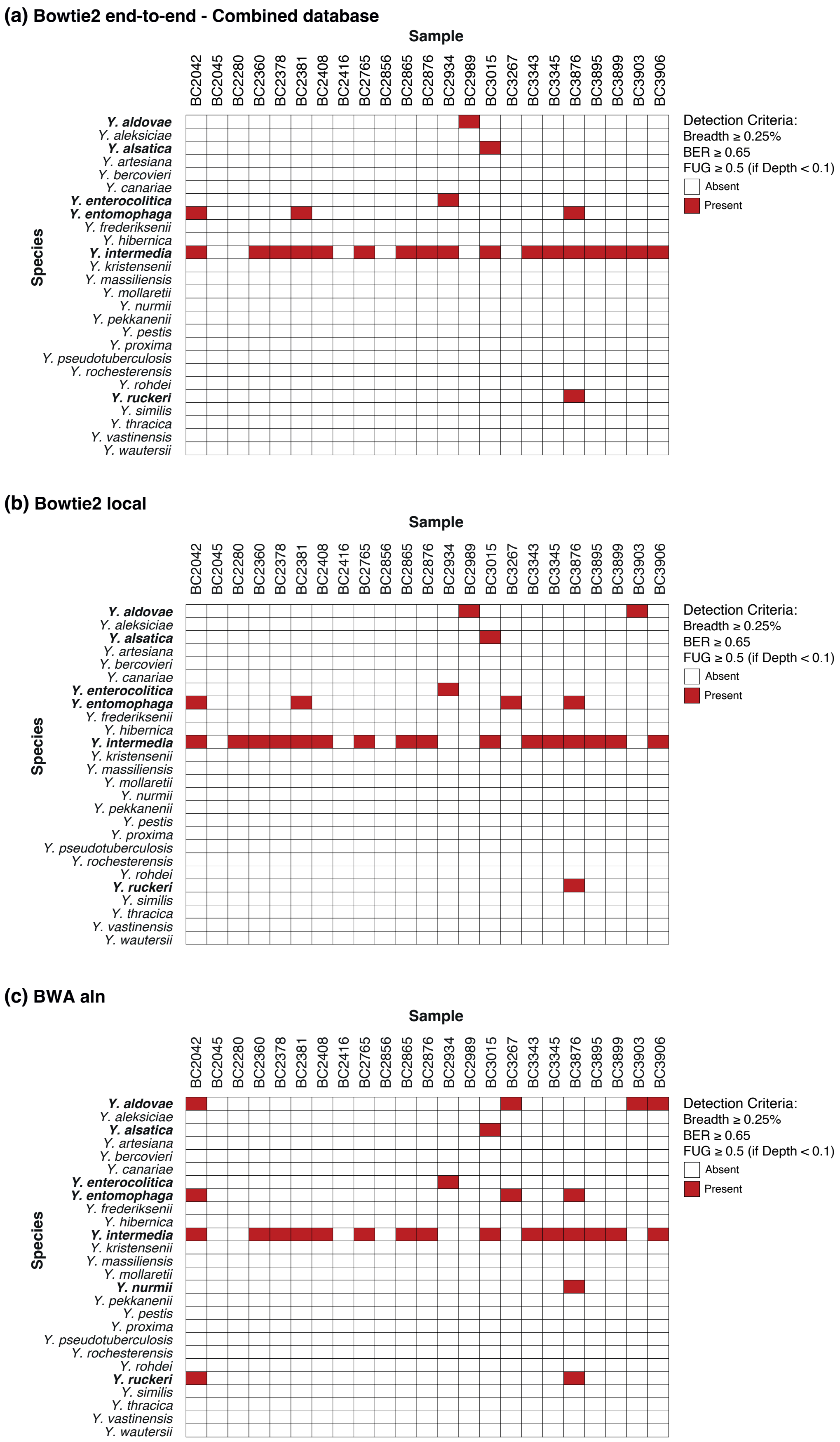

Figure S1. Identification of *Yersinia* species in 23 samples of Iberian desman using different approaches. (a) Identification with a combined database for all species using Bowtie2 in end-to-end mode. (b) Identification with single-species databases using Bowtie2 in default local mode. (c) Identification with single-species databases using BWA-aln.

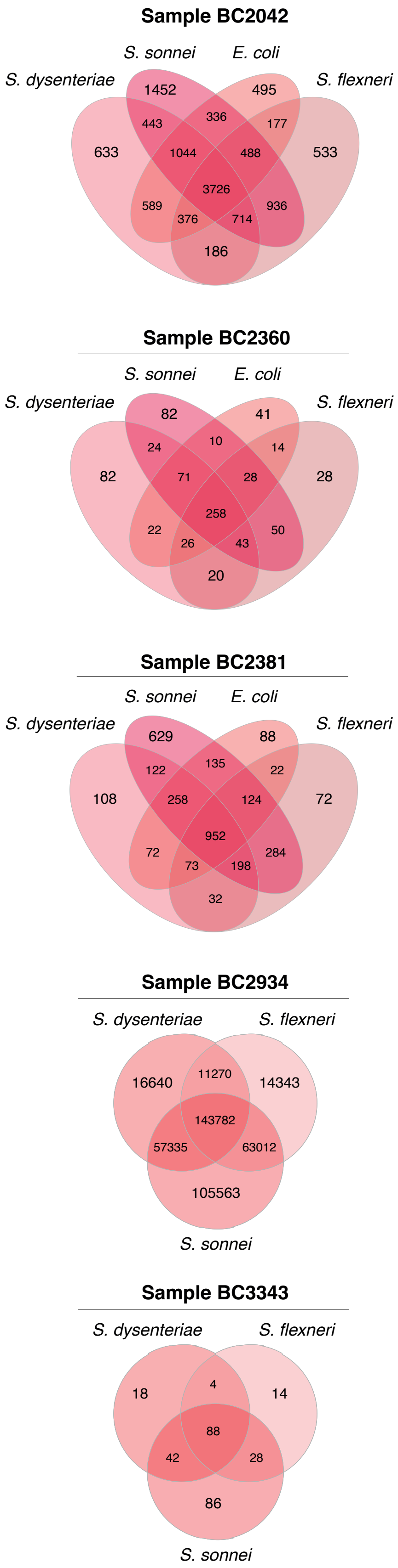

Figure S2. Venn diagram comparing reads assigned to species of the *Escherichia coli-Shigella* group in a single sample, showing shared reads (intersections) and unique reads (non-intersecting areas) for each species.

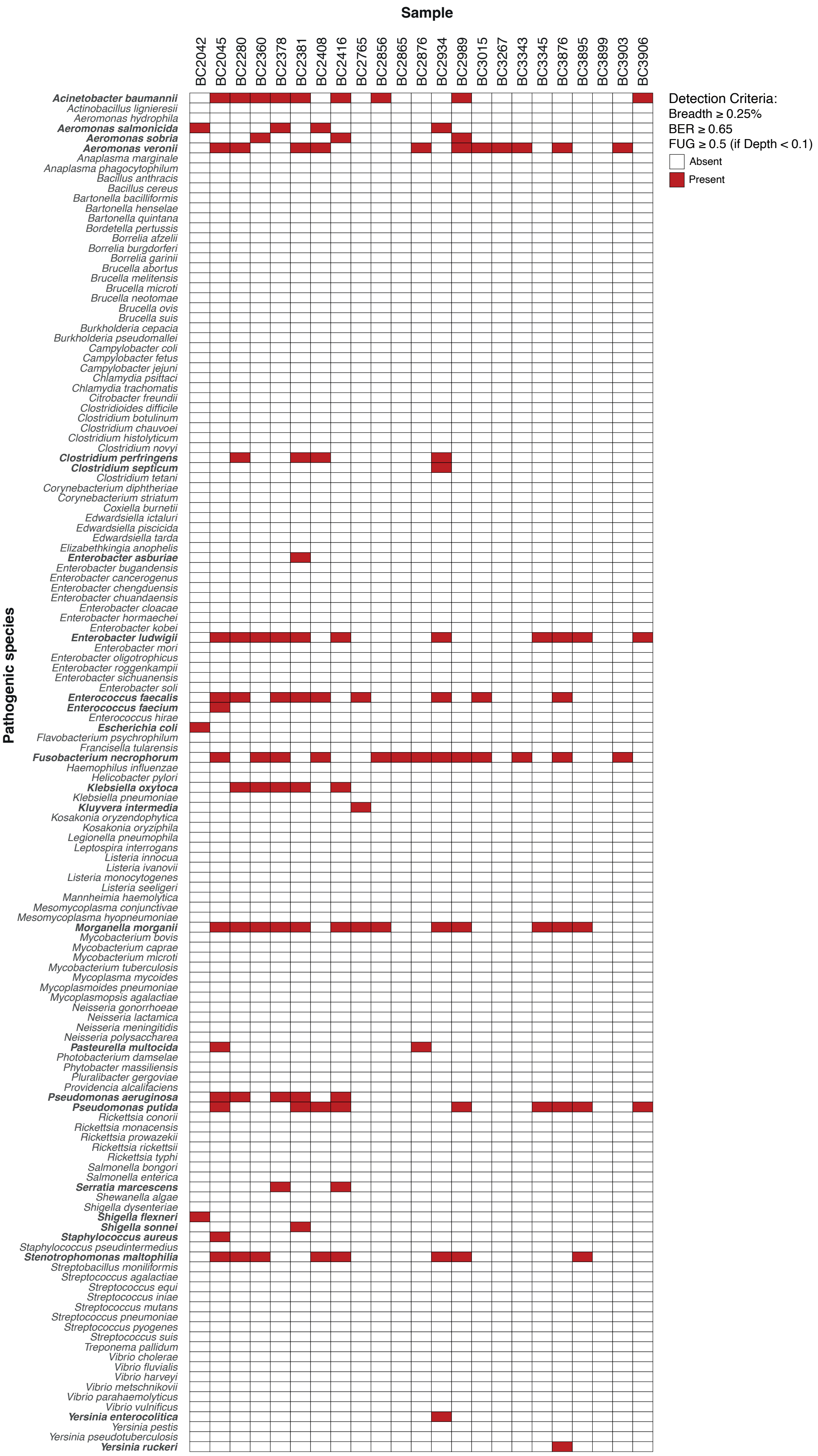

Figure S3. Identification of 136 bacterial species in 23 samples of Iberian desman using a combined database. Positive species are shown in bold for clarity.
